# Molecular karyotyping of Siberian wild rye (*Elymus sibiricus* L.) with oligonucleotide fluorescence *in situ* hybridization (FISH) probes

**DOI:** 10.1371/journal.pone.0227208

**Published:** 2020-01-17

**Authors:** Jihong Xie, Yan Zhao, Linqing Yu, Ruijuan Liu, Quanwen Dou

**Affiliations:** 1 Grassland Research Institute, Chinese Academy of Agricultural Sciences, Hohhot, China; 2 College of Grassland, Resource and Environmental Science, Inner Mongolia Agricultural University, Hohhot, China; 3 Key Laboratory of Crop Molecular Breeding, Qinghai Province, Northwest Institute of Plateau Biology, Chinese Academy of Sciences, Xining, China; 4 Laboratory of Adaptation and Evolution of Plateau Biota, Northwest Plateau Institute of Biology, Chinese Academy of Sciences, Xining, China; National University of Kaohsiung, TAIWAN

## Abstract

Siberian wild rye (*Elymus sibiricus* L.), an allotetraploid species, is a potentially high-quality perennial forage crop native to temperate regions. We used fluorescently conjugated oligonucleotides, representing ten repetitive sequences, including 6 microsatellite repeats, two satellite repeats, and two ribosomal DNAs, to characterize *E*. *sibiricus* chromosomes, using sequential fluorescence *in situ* hybridization and genomic *in situ* hybridization assays. Our results showed that microsatellite repeats (AAG)_10_ or (AGG)_10_, satellite repeats pAs1 and pSc119.2, and ribosomal 5S rDNA and 45S rDNA are specific markers for unique chromosomes. A referable karyotype ideogram was suggested, by further polymorphism screening, across different *E*. *sibiricus* cultivars with a probe mixture of (AAG)_10_, Oligo-pAs1, and Oligo-pSc119.2. Chromosomal polymorphisms vary between different genomes and between different individual chromosomes. In particular, two distinct forms of chromosome E in H genome were identified in intra- and inter-populations. Here, the significance of these results, for *E*. *sibiricus* genome research and breeding, and novel approaches to improve fluorescence *in situ* hybridization-based karyotyping are discussed.

## Introduction

Siberian wild rye (*Elymus sibiricus* L.) is widely distributed in the Northern hemisphere, with a particular preponderance in Sweden, northern Asia, Japan, and North America [1]. Due to its greater seedling vigor, cold tolerance, forage yield potential, and palatability (for livestock), it has been widely utilized as a forage crop [2, 3]. *E*. *sibiricus* is cultivated in the alpine pastures of the Qinghai-Tibet plateau of China as an important perennial and high-quality forage grass, and a few cultivars have been developed and popularized in this region. To improve the *E*. *sibiricus* cultivars genetically, several methods have previously been used, such as use of simple sequence repeat (SSR) markers, diversity evaluation, seed shattering variation, transcriptome profiling, and hybrid population construction [4–9]. Main targeted domestication traits, such as seed shattering and seed setting, still seriously hamper further utilization [7, 8] and genetic gain can be greatly improved by genomic selection. However, genome information for breeding of *E*. *sibiricus* remains insufficient. Understanding of the Triticeae genome structure can be greatly facilitated by comparative cytogenetic analysis, which can determine chromosomal collinearity and structure alteration with single-gene probes of wheat or barley [10, 11]. Furthermore, *de novo* sequencing and assembly in Triticeae species is difficult because of the large genomes. Dissecting the complex genome of Triticeae species can be accomplished using chromosome flow sorting, which dissects genomes into individual chromosomes using flow cytometric sorting [12].

As an allopolyploid species, *E*. *sibiricus* exhibits a genome constitution of StStHH (2n = 28), where St and H are derived from *Pseudoroegneria* (Neveski) LÖve and *Hordeum* L., respectively [13]. Using four repetitive sequences, including pAs1, (AAG)_10_, 5S rDNA, and 45S rDNA, as probes, molecular karyotyping of *E*. *sibiricus* was carried out through sequential fluorescence *in situ* hybridization (FISH) and genomic *in situ* hybridization (GISH) assays [14]. However, presence of limited hybridization sites made it difficult to distinguish between some chromosomes of the St and H genomes, and the limited *E*. *sibiricus* samples used in this study were insufficient to infer a referable karyotype for *E*. *sibiricus*. Many repetitive sequences have been characterized in Triticeae species, and these can be used for chromosomal identification purposes across different species or genomes [15, 16]. Furthermore, oligonucleotide FISH probes can be used instead of repetitive sequences and are commonly used to distinguish between wheat and rye chromosomes [17, 18]. FISH using oligonucleotide probes is a more convenient and stable method than conventional FISH analysis [18].

In this study, multiple oligonucleotides representing different repetitive sequences were characterized and screened in *E*. *sibiricus* using the FISH method. A robust molecular karyotype of *E*. *sibiricus* was generated by a one-step FISH method. In addition, intra-population and inter-population chromosomal polymorphisms have been described and discussed.

## Materials and methods

### Plant materials

Four *E*. *sibiricus* cultivars were used in this study. Qingmu No. 1 and Tongde were developed in Qinghai, whereas Nongmu and Chuancao No. 2 were developed in Inner Mongolia and Sichuan in China, respectively. The original populations of these four cultivars were geographically distant. All seeds are available in the National Forage Germplasm Mid-term Bank in Grassland Institute, Chinese Academy of Agricultural Sciences, China. More than 10 individuals were used for detailed karyotyping of Tongde and Nongmu. Two individuals of Tongde and one individual each of the others were randomly selected to test karyotype variability using one-step FISH.

### Probe DNA preparation and labeling

Six microsatellites, two satellite sequences, and two ribosomal DNAs were used for karyotyping. The six microsatellites used were oligonucleotides (AAG)_10_, (AGG)_10_, (AAC)_10_, (ACT)_10_, (CAT)_10_, and (CAG)_10_. The two satellite sequences used were pAs1 [19] and pSc119.2 [20], and the two ribosomal DNAs used were 5S and 45S rDNA. The designated oligonucleotides pAs1-1 plus pAs1-2 and 5Sg representing pAs1 and 5S [17], respectively, and designated oligonucleotides Oligo-pTa71-2 and Oiogo-pSc119.2–1 plus Oligo-pSc119.2–2 representing 45S rDNA and pSc119.2 [18], respectively, were used to generate FISH probes. Thus, all oligonucleotides were end-labeled using either fluorescein amidite (FAM; green) or carboxytetramethylrhodamine (TAMRA; red) (Sangon Biotech Co., Ltd., Shanghai, China). Additionally, (AAG)_10_ was labeled at both the 3' and 5' ends with FAM and TAMRA simultaneously and subsequently used for one-step karyotyping. To distinguish between St and H genomes in *E*. *sibiricus*, GISH assays were conducted, using labeled genomic DNA of *Hordeum bogdanii* (2n = 14, HH genome) and *Pseudoroegneria stipifolia* (2n = 14, StSt genome), as described by Dou *et al*. [14]. For preparing GISH probes, genomic DNAs of *Hordeum bogdanii* and *P*. *stipifolia*, were fragmented by autoclaving at 120°C for 2 min before labelling. DNAs of *H*. *bogdanii* were labelled with fluorescein-12-dUTP, whereas those of *P*. *stipifolia* were labelled with tetramethy1-rhodamine-5-dUTP, using the random primer labeling method. To increase labeling efficiency, the duration of random primer labeling reaction was extended for 24 h.

### Slide preparation

Seeds of *E*. *sibiricus* were germinated on moist filter paper, in petri dishes, at 26°C. Excised root tips, approximately 1–2 cm long, were placed in a chamber, treated with nitrous oxide gas (10 atm) for 2 h, as described by Kato [21], and fixed in 3:1 (v/v) 100% ethanol: glacial acetic acid. Each root tip was squashed in a drop of 45% acetic acid and observed using a phase contrast microscope (Olympus Bx40). The slides containing metaphase chromosomes were frozen at **−**80°C for more than 30 min and cover slips were removed promptly with a razor. Thereafter, the slides were dried at 26°C for further processing.

### *In situ* hybridization

The prepared slides were denatured in 0.2 M NaOH, in 70% ethanol, at 25 ± 2°C for 10 min, rinsed in 70% cold ethanol (pre-cooled at **−**20°C) for approximately 30 min, and air dried. A total of 10 μL of hybridization mixture, prepared by adding 50% de-ionized formamide, 50% dextran sulfate, 2 × SSC (0.3 M NaCl and 0.03 M trisodium-citrate), 1 μg/μL denatured salmon sperm DNA, and 10 ng of probe, was added to each slide. The hybridization mixture, with oligonucleotide probes, was placed directly onto the denatured preparation. The hybridization mixture, with labeled *H*. *bogdanii* and *P*. *stipifolia* genomic DNA, was denatured in boiling water for 5 min. Hybridization was conducted overnight at 37°C in a humidified chamber. The sequential hybridization procedure was as follows: after photographing the first probing, the cover slips were removed, and the slides were washed in 2× SSC for 20 min at room temperature and briefly dried; the mixture for the second hybridization was applied to the slide, without denaturation. The subsequent steps were the same as those for the first hybridization.

### Microphotometry

Chromosomes were counterstained with 4', 6-diamidino-2-phenylindole. Images were captured with a cooled charge-coupled device camera (Photometrics CoolSNAP) using a fluorescence microscope (Leica) and were processed with the Meta Imaging System (Universal Imaging Corporation).

## Results

**Fig 1:**
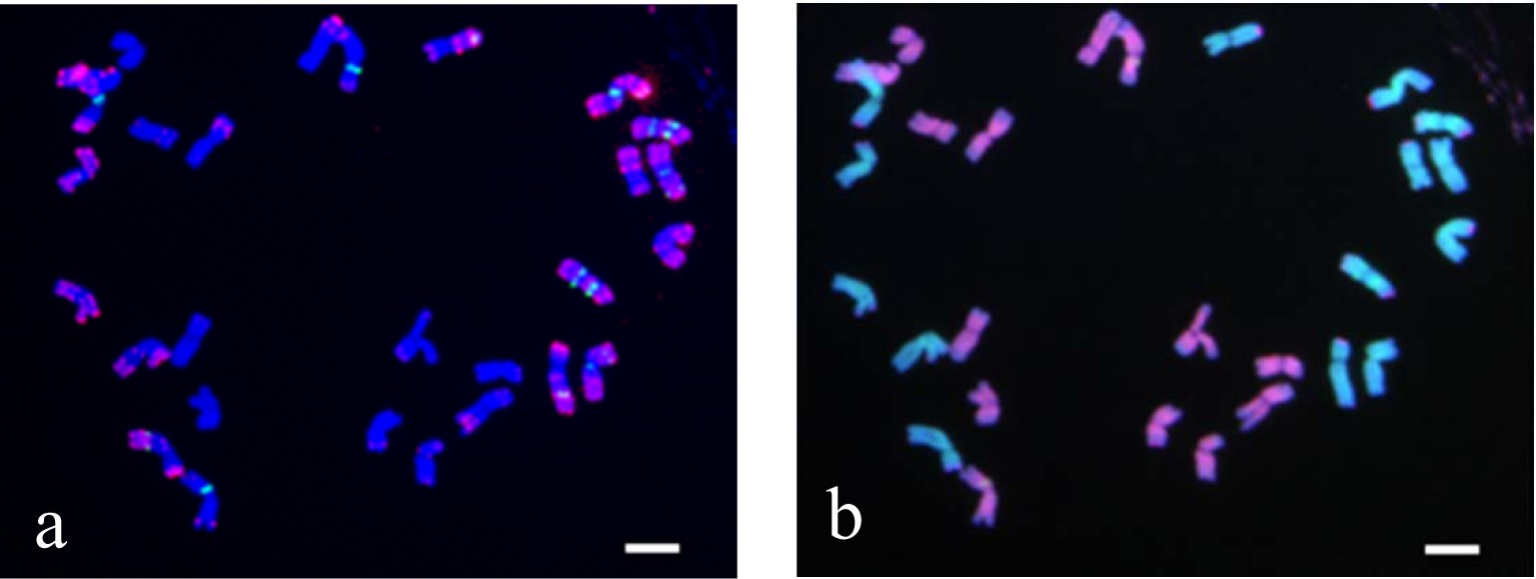
Sequential FISH and GISH patterns of *Elymus sibiricus* ‘Nongmu’. a: FISH pattern probed with (AAG)_10_ (green) and Oligo-pAs1 (red); b: Sequential GISH pattern probed with *Hordeum bogdanii* (green) and *Pseudoroegneria stipifolia* (red). Scale bar = 10 μm.

Due to the allopolyploidy observed in *E*. *sibiricus*, we employed sequential FISH and GISH for chromosome identification. First, *E*. *sibiricus* chromosomes were identified by FISH using two different repetitive sequences; thereafter, each chromosome was assigned to the St or H genome by GISH (Fig 1). Since the repetitive sequence, Oligo-pAs1, produced far more hybridization sites in H and St genomes, chromosomal localization of other repetitive sequences was performed using Oligo-pAs1 as a reference marker (Fig 1).

**Fig 2:**
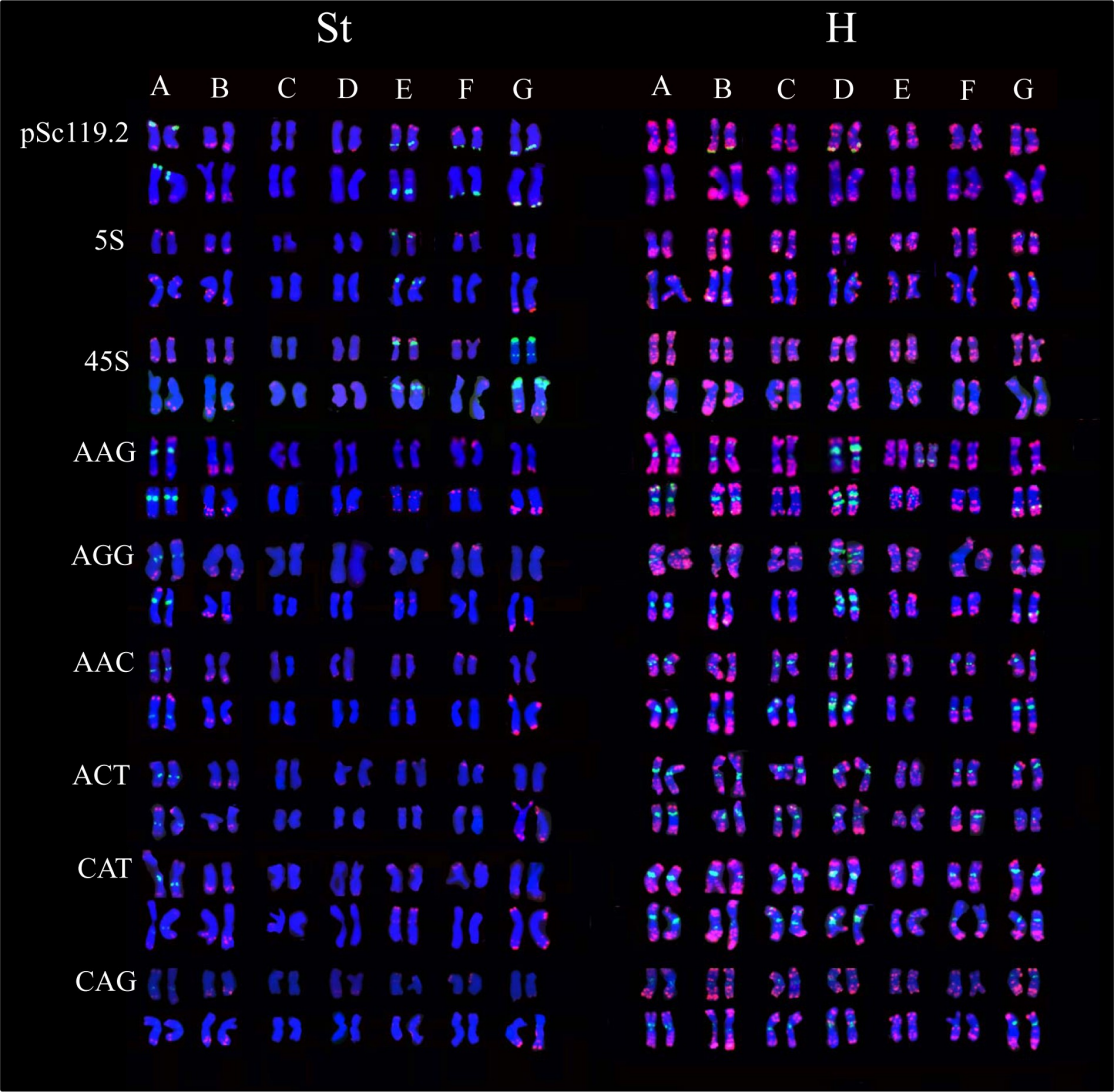
**Molecular karyotyping of the two cultivars of *Elymus sibiricus* combined with Oligo-pAs1 (red) and different repetitive sequences (green)**. The upper panel shows the karyotype for Tongde and the lower for Nongmu, for each characterization.

To develop a reference karyotype for all *E*. *sibiricus* cultivars or accessions, we selected two geographically distant cultivars, Tongde and Nongmu, for detailed karyotyping, using different combinations of repeats. Thus, molecular karyotyping of two cultivars of *E*. *sibiricus* was performed using a combination of Oligo-pAs1 and nine other different repetitive sequences (Fig 2). The results showed the karyotype formulas of *E*. *sibiricus* as 4”m+3”sm and 4”m+3”sm in both the St and H genomes. Since the homoeologous group could not be determined in these chromosomes, we arbitrarily designated well-marked chromosomes in each genome as A-G, by their arm ratio and relative length (S1 Table and Fig 2). In both Tongde and Nongmu, the repetitive sequence, Oligo-pAs1, produced stronger and richer hybridizations in the H genome than in the St genome. It facilitated the separation of H chromosomes from St chromosomes. The chromosomes of the H genome could be adequately identified by the Oligo-pAs1 FISH pattern. However, polymorphic hybridizations of Oligo-pAs1 in intra- and inter cultivars were observed, especially on chromosome A and B in the H genome (Fig 2). *E*. *sibiricus* ‘Tongde’ showed a polymorphic Oligo-pAs1 hybridization site in the pericentric region of A chromosome, and both cultivars showed a polymorphic Oligo-pAs1 hybridization site in the interstitial regions of the long arm of chromosome A. Both cultivars showed a polymorphic hybridization site in the pericentric region of the long arm of B chromosome. A stable Oligo-pAs1 hybridization site was observed around the centromere of the long arm of C chromosome in both cultivars, but variation in hybridization intensity at this site, was detected in Nongmu. Oligo-pAs1 hybridization sites, around the centromeres of the short arms of chromosomes D and E, and around the centromeres of the long arms of chromosomes F and G, were detected in both cultivars. A reduced frequency of Oligo-pAs1 hybridization was observed in St chromosomes. Further, Oligo-pAs1 hybridization could be detected on three to four pairs of St chromosomes in both cultivars. B chromosomes were accurately detected with Oligo-pAs1 hybridization in the interstitial regions of the long arms in both cultivars, although variations in hybridization intensity were observed in both intra- and inter-cultivars. Oligo-pAs1 hybridization sites were identified at both ends of A chromosome in both cultivars; however, polymorphism in the form of presence or absence of hybridization in one end, was frequently detected in Nongmu. The Oligo-pAs1 hybridization site, at the end of the long arm of D chromosome, was more frequently detected in Tongde than in Nongmu. This was true for Oligo-pAs1 hybridization at the end of the short arm of E and F chromosomes. Polymorphic distribution patterns of Oligo-pAs1 hybridization were observed at both ends of G chromosomes in intra- and inter-cultivars. However, Oligo-pAs1 hybridization of G chromosomes in Nongmu showed higher hybridization intensity than in Tongde. Four stable Oligo-pSc119.2 hybridization sites were detected in St chromosomes in both cultivars, but only two terminal hybridization sites in the H genome were detected in Nongmu. One stable 5S rDNA site was revealed in one pair of St chromosomes (E chromosome) in both cultivars. Four to five 5S rDNA sites were detected in H genome chromosomes. The hybridization of 5S rDNA in H chromosomes was faint and polymorphic between two cultivars, except for one interstitial site on the short arm on chromosome G that was stronger and more distinct. Three 45S rDNA sites were identified in two St chromosomes (chromosome D and G) in both cultivars, while no apparent 45S rDNA sites were detected in H chromosomes. The hybridization pattern of microsatellites (AAG)_10_ and (AGG)_10_ appeared to co-localize. Only one (AAG)_10_ or (AGG)_10_ was strongly identified in one pair of St chromosomes (Chromosome A) in both cultivars. However, distinct hybridization of (AAG)_10_ or (AGG)_10_ was detected on four to five H genome chromosomes in both cultivars. The hybridization of (AAG)_10_ or (AGG)_10_ was stably detected in Tongde, while those in A and E chromosomes of the H genome were variable in Nongmu. This revealed that hybridization patterns of (AAG)_10_ or (AGG)_10_ are extremely informative for chromosome identification of the H genome. Co-localized distribution of (AAC)_10_, (ACT)_10_, and (CAT)_10_ was also observed in both St and H genome chromosomes. One pair of St chromosomes (chromosome A) and almost all H chromosomes exhibited (AAC)_10_, (ACT)_10_, or (CAT)_10_ hybridization. However, the distribution patterns between different H chromosomes were less varied than those of (AAG)_10_ or (AGG)_10._ Furthermore, variable (AAC)_10_, (ACT)_10_, or (CAT)_10_ hybridization was not observed across different individuals in each cultivar. However, polymorphic distribution of repeats in C and D chromosome were revealed in H genome between Tongde and Nongmu. Faint and polymorphic hybridizations of (CAG)_10_ were observed on most H chromosomes and few St chromosomes in the two cultivars. The distribution of (CAG)_10_ was mainly around the centromere in most H chromosomes and conserved between the two cultivars. However, the hybridization intensity of (CAG)_10_ of the H genome was less in Tongde than in Nongmu. The hybridization of (CAG)_10_ in St chromosome in Nongmu was barely detectable. The faint signals and the minimal variability in distribution pattern made it less reliable as a chromosomal marker.

**Fig 3:**
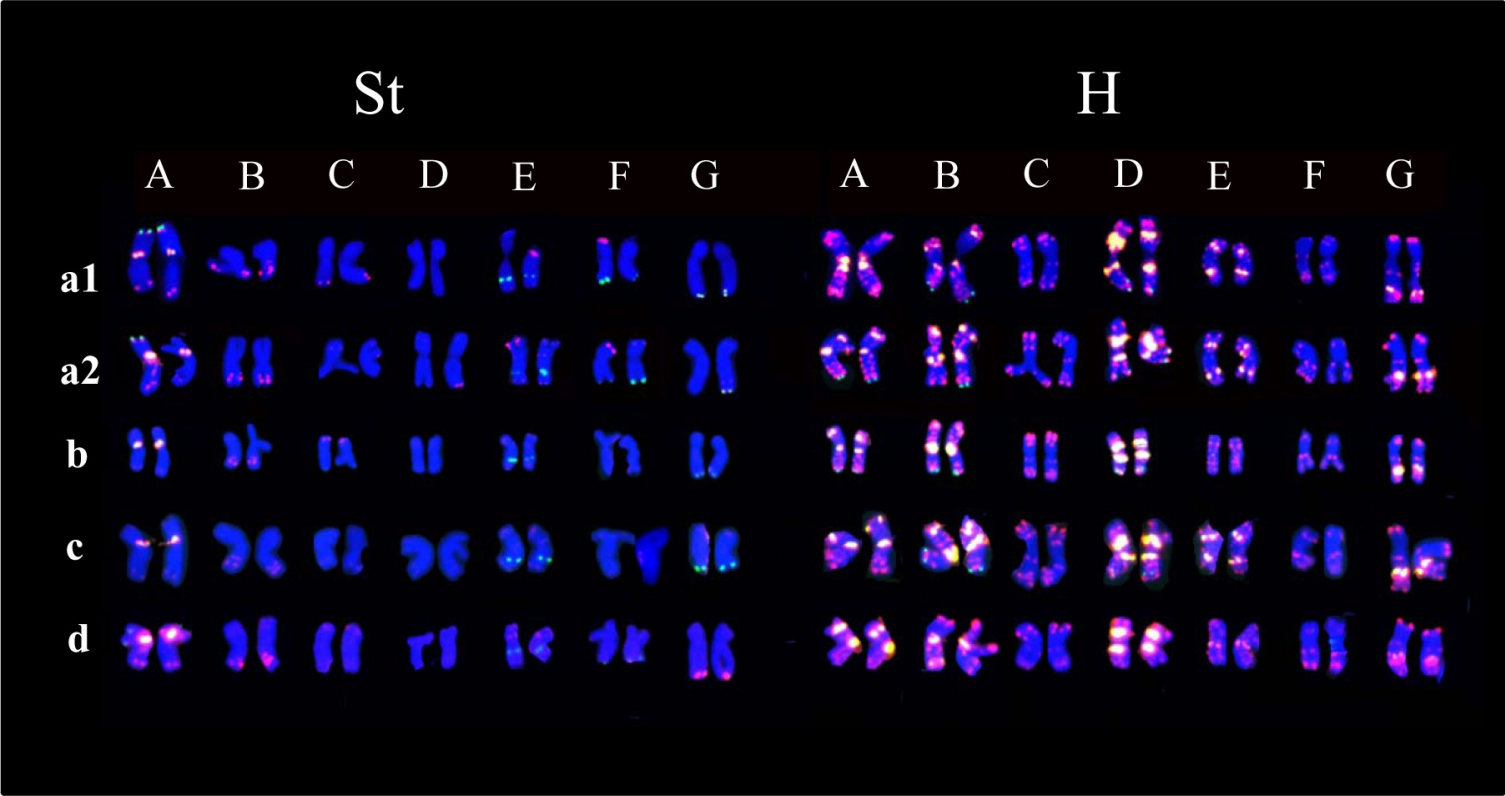
**Karyotyping of four *Elymus sibiricus* cultivars, probed with Oligo-pSc119.2 (green), (**AAG**)**_**10**_ **(yellow), and Oligo-pAs1 (red).** A1 and A2: Two different samples from Tongde; B: Chuancao No. 2; C: Qingmu No. 1; and D: Nongmu.

Physical mapping of 10 repetitive sequences on *E*. *sibiricus* chromosomes showed that the repeats Oligo-pAs1, (AAG)_10_ and (AGG)_10_, Oligo-pSc119.2, 5S rDNA, and 45S rDNA were reliable chromosomal markers for differentiating between individual chromosomes. Notably, a combination of distribution patterns of these sequences was expected to make it more convenient to classify each chromosome. Thus, a one-step FISH method was developed to identify chromosomes in *E*. *sibiricus* by using combinations of Oligo-pAs1, Oligo-pSc119.2, and (AAG)_10_ as probe. To test the stability of the chromosomal markers or karyotype variability among different *E*. *sibiricus* varieties, besides Tongde and Nongmu, two other distant cultivars Chuancao No. 2 and Qingmu No. 1 were examined using the one-step FISH method. Conserved karyotypes were unambiguously obtained across the different cultivars (Fig 3). However, significant variations, in the presence and size of the repeat sequences, were observed in intra- and inter-cultivars. Karyotyping with a combination of three repeats revealed polymorphic homologous chromosomes E and F of St genome in some samples (Fig 3A1), polymorphic homologue chromosomes E, F, and G of St genome, and B chromosome of H genome in other samples in Tongde (Fig 3A2), which showed the heterozygosity of these samples. Polymorphic hybridizations were detected in chromosomes A, E, F, and G of the St genome, and frequently observed chromosomes were A, B, E, and F of the H genome, across different cultivars. Two distinct variants of E chromosome of H genome were revealed among different cultivars (Fig 3). Since two variants were frequently detected among different individuals in both Tongde and Nongmu (Fig 2), it is possible that they co-existed in all *E*. *sibiricus* populations universally.

**Fig 4:**
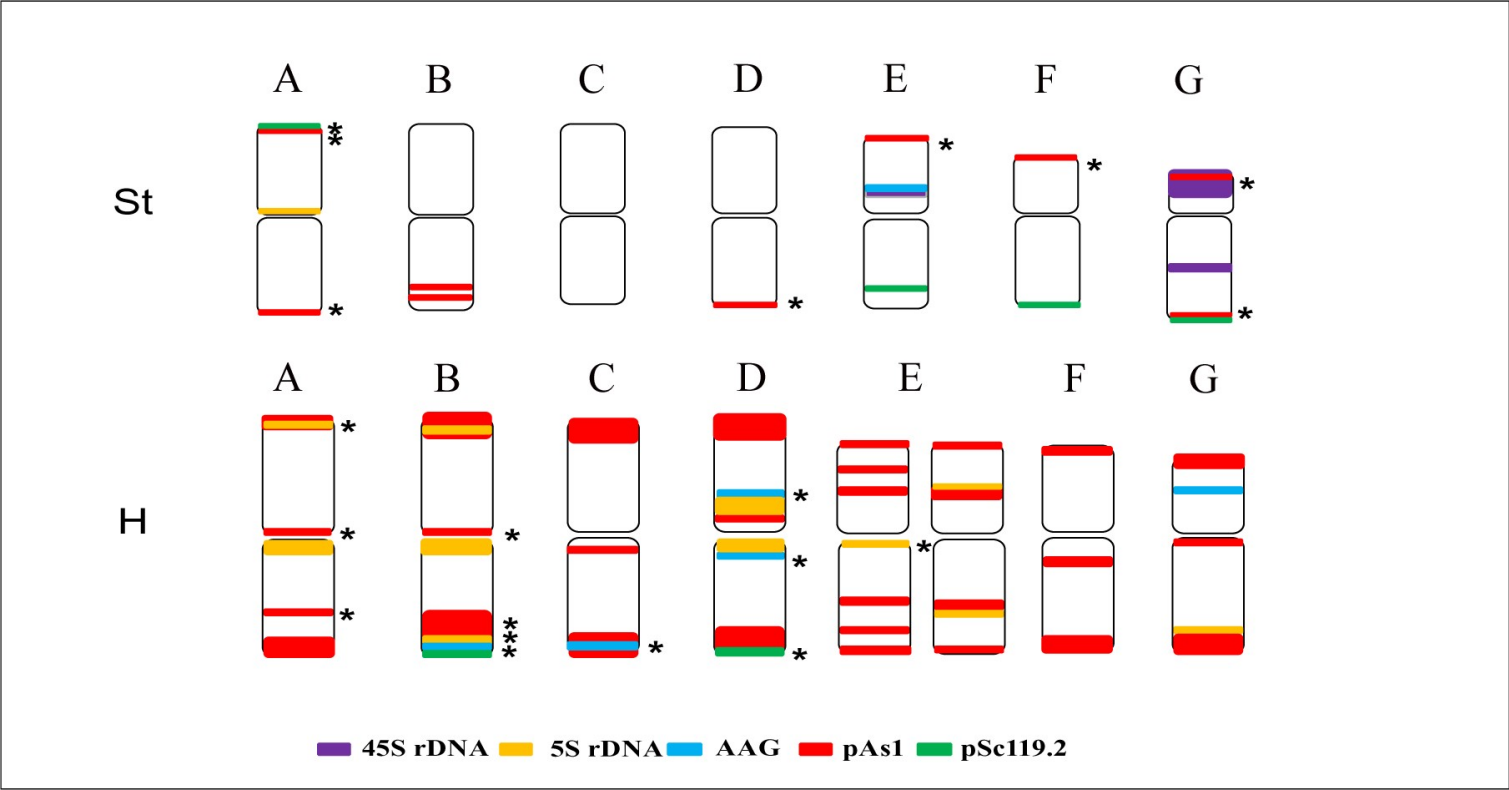
Ideogram of FISH-banded chromosomes of *Elymus sibiricus*. The black star next to the FISH signal indicates the polymorphisms.

Based on the distribution pattern of each repetitive sequence in the two *E*. *sibiricus* cultivars and the polymorphic distribution patterns of the selected repeats, an ideogram of a standard karyotype was constructed, as presented in Fig 4. A brief description of the FISH pattern of *E*. *sibiricus* is as follows:

In the St genome, chromosome A is distinct, with the (AAG)_10_ or (AGG)_10_ hybridization site in the pericentric region of the short arm. Chromosome B has two Oligo-pAs1 hybridization sites in the interstitial region of the long arm. Chromosome C and D are distinguished from the other St chromosomes with no distinct hybridizations. Chromosome D is distinguishable from C with a larger ratio of long arm to short arm. Chromosome E strongly exhibits an interstitial Oligo-pSc119.2 hybridization site on the long arm, and 5S rDNA and 45S rDNA site in the short arm. Chromosome F presents the second largest arm ratio, carrying a terminal Oligo-pSc119.2 hybridization site on the long arm, in most instances. Chromosome G shows the largest arm ratio and carries the 45S rDNA site on both short and long arms.

The large size, metacentric shape, and abundant Oligo-pAs1 hybridization sites enable chromosomes of the H genome to be distinguished from the chromosomes of the St genome. In the H genome, chromosomes A and B are the most highly polymorphic in the intra- or inter populations. Chromosome B carries more dispersed Oligo-pAs1 hybridizations in the long arm than chromosome A. Chromosome C has no (AAG)_10_ or (AGG)_10_ hybridization sites, but exhibits a strong Oligo-pAs1 hybridization signal in the sub-telomeric region of the short arm and the pericentric region of the long arm, which is distinct from the others. Chromosome D is exclusively identified by a strong (AAG)_10_ or (AGG)_10_ in the interstitial region of the short arm and in the pericentric region of the long arm, respectively. Two distinct variants of chromosome E were identified in intra- or inter-populations. One variant carries dispersed Oligo-pAs1 hybridization sites nearly all over the chromosome and a faint (AAG)_10_ or (AGG)_10_ signal around the centromere in most cases, whereas the other variants present two strong (AAG)_10_ or (AGG)_1o_ hybridization signals in the interstitial regions of both the short and long arms. Chromosome F shows similar FISH pattern to C, but is distinct from C because it exhibits less Oligo-pAs1 hybridizations in the short arm and a larger arm ratio. Chromosome G is distinguishable from the others with less Oligo-pAs1 hybridizations in the short arm and a prominent (AAG)_10_ or (AGG)_1o_ hybridization site in the interstitial region of the long arm.

## Discussion

In the previous study [14], a molecular karyotype of *E*. *sibiricus* was suggested by adopting a three-step sequential FISH and GISH technique; first, using 5S rDNA and 45S rDNA probes, thereafter, using (AAG)_10_ and Afa family repetitive sequence probes, and finally using St and H genome DNA probes. Using this method, three chromosomes of the St genome and five chromosomes of the H genome were identified, and the others were characterized using chromosome morphology, such as arm ratio and relative length. In the present study, a detailed molecular cytogenetic karyotype was described using 10 repeats, among which 5S rDNA, 45S rDNA, pAs1, and Oligo-pSc119.2 were informative enough to distinguish each chromosome exclusively. Additionally, the DNA FISH probes in a previous study were produced by PCR amplification (5S rDNA and Afa family repetitive DNA) and clone DNA (45S rDNA) [14]. However, the FISH probes used in this study are all oligonucleotide probes. Fluorescently labeled synthetic repetitive DNA probes are helpful in FISH in suspension and chromosome flow sorting [22]. Although the (AAC)_10,_ (CAT)_10_, and (ACT)_10_ repeats are not informative for distinguishing between chromosomes, due to less polymorphism across chromosomes, their high hybridization intensity for the major chromosomes of the H genome makes them useful as chromosomal markers for chromosome flow sorting [12]. In this study, the developed molecular karyotype will provide a basic tool to explore and dissect the genome of *E*. *sibiricus* further, using comparative cytogenetics or flow sorting.

Genetic variation in *E*. *sibiricus* was evaluated in previous studies using molecular markers, such as ISSR [23], SSR [24], Gliadin [6], and Scot [25]. These results showed that a little more than 50% genetic variation is found within populations, while the rest is observed among populations. In our study, chromosome polymorphisms were detected not only in between different cultivar populations, but also within populations of the same cultivar. Moreover, the result showed that the polymorphisms varied among different chromosomes. The H genome is more variable than the St genome. In the St genome, chromosomes A, B, E, and G were more conserved than the others. In the H genome, chromosomes A and B were most variable, whereas chromosomes C, D, F, and G were more conserved. Additionally, heterozygote karyotypes were revealed in individuals of *E*. *sibiricus* cultivars, such as in Tongde and Nongmu. Though *E*. *sibiricus* is regarded as a self-pollinating species, high intra-population variation and high frequency of heterozygous karyotype indicates that the out-crossing rate, to some extent, can be maintained within the population. In particular, two distinct forms of chromosome E of H genome were identified across different cultivars and different individuals in the same cultivar. This suggests that lack of recombination between these chromosomes may lead to apparent molecular cytogenetic diversification. The biological significance of the two distinct chromosomes is unknown. In this study, frequent chromosome polymorphism and heterozygosity were revealed within the population of the cultivar. Since homozygosity or pure line selection may bring about high seed setting for self-pollinating species, *E*. *sibiricus* seed setting may be improved by a homozygous genotype and pure line selection.

Karyotype variation diversity varies by geographical region and was reported in Triticeae species, such as *Triticum araraticum* Jakubz. and *Triticum dicoccoides* Korn [26, 27]. A unique spectrum of translocations was seen in each geographical region [26]. In this study, no apparent chromosomal rearrangements were detected among the four geographically distant cultivars. Detailed comparison of the Tongde and Nongmu karyotypes showed that Nongmu had a higher hybridization intensity of Oligo-pAs1 in the G chromosome of St genome and those of (CAG)_10_ in the H genome chromosomes were distinct from Tongde. Due to the high frequency of chromosomal polymorphism in the intra-populations, limited samples for other cultivars, and limited *E*. *sibiricus* accessions used in this study, region specific chromosome variations in *E*. *sibiricus* need to be further studied.

FISH probes are tagged using methods such as nick-translation, addition of random primers, or PCR-based methods. These approaches are laborious and time-consuming and oligonucleotide probes can be used to replace repetitive sequences [17, 18]. Oligonucleotides can easily be labeled by the addition of fluorescein nucleotides at the 3' or 5' end. These labeled probes can be acquired from commercial suppliers. The oligonucleotide probes used in this study were shown to be able to accurately substitute the microsatellite and other tandem repetitive sequences. Chromosome identification can be greatly improved by using a probe mixture, containing multiple probes labeled with different fluorescein molecules [28]. In this study, we used two kinds of fluorescein dyes, namely FAM; green and TAMRA; red. However, simultaneous labeling of (AAG)_10_ at the 3' and 5' ends by FAM and TAMRA, produced a fluorescent signal (yellow), which facilitated chromosome identification. This observation suggests that a wide array of oligonucleotide probes can be produced with different fluorescein combinations using other fluorescein dyes, such as Coumarin (blue).

Abundant information about the frequency of repetitive sequences is available for the Triticeae species. The repetitive sequences utilized in this study, have been well documented by previous studies on other Triticeae species. Next generation sequencing (NGS) helps generate sequence information for large genomes. Even at a relatively low coverage rate (~1%), NGS can be instrumental in generating multiple repeats [29]. Oligonucleotide FISH probes can be produced using NGS data [30]. Thus, we postulate that chromosome resolution can be greatly improved by utilizing NGS, coupled with the oligonucleotide-based FISH probe technique.

## Supporting information

S1 Table Basic Karyotype features of the *E*. *sibiricus*. (DOCX)
