# Supplementary material for: Molecular karyotyping of Siberian wild rye (Elymus sibiricus L.) with oligonucleotide fluorescence in situ hybridization (FISH) probes
Source: PLoS One. 2020 Jan 17;15(1):e0227208. doi: 10.1371/journal.pone.0227208 (PMC6968859; doi:10.1371/journal.pone.0227208)
Supplement: S1 Table — (DOCX) [file pone.0227208.s001.docx]

S1 Table. Basic Karyotype features of the *E. sibiricus*

| Genome | Chromsome No. | Arm ratio | Relative length | Chromosome  type |
| --- | --- | --- | --- | --- |
| St | A | 1.19±0.08 | 0.17±0.03 | m |
|  | B | 1.12±0.09 | 0.14±0.02 | m |
|  | C | 1.09±0.06 | 0.13±0.02 | m |
|  | D | 1.21±0.09 | 0.13±0.01 | m |
|  | E | 1.27±0.10 | 0.13±0.03 | sm |
|  | F | 1.29±0.13 | 0.13±0.03 | sm |
|  | G | 2.15±0.12 | 0.17±0.03 | sm |
| H | A | 1.08±0.04 | 0.16±0.01 | m |
|  | B | 1.18±0.10 | 0.16±0.03 | m |
|  | C | 1.12±0.04 | 0.14±0.02 | m |
|  | D | 1.10±0.05 | 0.14±0.01 | m |
|  | E | 1.17±0.07 | 0.11±0.00 | sm |
|  | F | 1.18±0.14 | 0.12±0.01 | sm |
|  | G | 1.76±0.11 | 0.14±0.02 | sm |

Arm ratio=Long arm/Short arm; Relative length= Chromosome length/total chromosome length of the respective St or H genome.
